# Trends and Characteristics of Nursing Home Obesity Rates, 2011–2021

**DOI:** 10.1093/geroni/igaf122.402

**Published:** 2025-12-31

**Authors:** Hye-Young Jung, Hyunkyung Yun, Jihoon Park

**Affiliations:** Joan and Sanford I. Weill College of Medicine, Cornell University, New York, New York, United States; Brown University, Providence, Rhode Island, United States; McDaniel College, Providence, Rhode Island, United States

## Abstract

Nursing home (NH) residents have historically had high rates of obesity. However, recent trends and NH factors related to them are understudied. We described national trends in obesity rates among NH residents from 2011 to 2021 and examined facility characteristics associated with them in 2021. NH facility characteristics were obtained from LTCFocus. Obesity rates were defined as the proportion of residents with body mass index ≥ 35. The sample included 16,712 NHs in 49 states. Resident obesity rates increased from 26.5% in 2011 to 30.5% in 2021, a 15% relative increase, with substantial geographic variation. All states experienced increases with Idaho exhibiting the largest increase (11.8 percentage points (pp); from 29.1% to 40.9%) and Louisiana experiencing the smallest (0.5 pp; from 28.4% to 28.9%). Compared to NHs in the lowest quartile of obesity rates, residents of those in the highest quartile were younger on average (74.9 vs. 78.0; p < 0.001), had a lower proportion of non-Hispanic White residents (19.0% vs. 30.5%; p < 0.001), and a higher share of Medicaid-covered residents (65.3% vs. 61.9%; p < 0.001). They also exhibited lower occupancy rates (72.1% vs. 77.8%; p < 0.001), were more likely to be part of a multi-facility chain (65.1% vs. 53.1%; p < 0.001), and to be in a rural area (37.5% vs. 14.2%: p < 0.001). Our findings indicate continued increases in obesity rates among NH residents with wide geographic variation, and potential disparities associated with race/ethnicity, rurality, and income. Future research is warranted to examine the relationship between NH characteristics and care quality for residents with obesity.

